# Anti-angiogenic drug loaded liposomes: Nanotherapy for early atherosclerotic lesions in mice

**DOI:** 10.1371/journal.pone.0190540

**Published:** 2018-01-16

**Authors:** Isabel Pont, Aracely Calatayud-Pascual, Alicia López-Castellano, Elena P. Albelda, Enrique García-España, Luis Martí-Bonmatí, Juan C. Frias, M. Teresa Albelda

**Affiliations:** 1 Instituto de Ciencia Molecular, Universidad de Valencia, Valencia, Spain; 2 Departamento de Fisiología, Farmacología y Toxicología, Universidad Cardenal Herrera-CEU, CEU Universities, Alfara del Patriarca, Spain; 3 Departamento anestesiología, Hospital Lluís Alcanyís, Xàtiva, Valencia, Spain; 4 Grupo de investigación biomédica en imagen, Hospital Universitario y Politécnico La Fe, Valencia, Spain; 5 Departamento de Ciencias Biomédicas, Universidad Cardenal Herrera-CEU, CEU Universities, Alfara del Patriarca, Spain; Medstar Washington Hospital Center, UNITED STATES

## Abstract

Fumagillin-loaded liposomes were injected into ApoE-KO mice. The animals were divided into several groups to test the efficacy of this anti-angiogenic drug for early treatment of atherosclerotic lesions. Statistical analysis of the lesions revealed a decrease in the lesion size after 5 weeks of treatment.

## Introduction

Over the past decade, nanoparticles have been explored for both drug delivery and imaging applications. The term theranostic was coined to describe this emerging technology, where an imaging moiety is combined with a therapeutic effect within a single nanoparticle. For example, different nanoparticles have been employed for the detection and treatment of cancer. However, the use of nanoparticles for the treatment of atherosclerotic plaques remain scarce [1,2].

In our approach, liposomes were chosen as carriers based on the flexibility that these types of nanoparticles offer during their preparation. Their size can be easily modified allowing the incorporation of several imaging agents and homing molecules to selected targets into the phospholipid bilayer [3]. In addition, their hollow core enables incorporation of lipophilic or hydrophilic therapeutic drugs to generate a multimodal theranostic agent.

**Fig 1:**
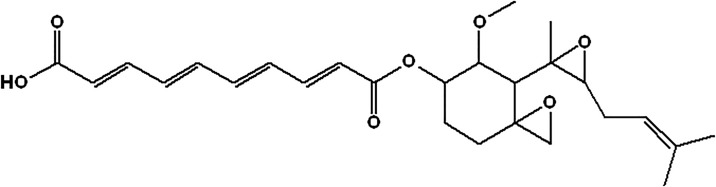
Chemical structure of fumagillin.

Fumagillin is a mycotoxin produced by Aspergillus fumagatus (Fig 1). It is a selective inhibitor of endothelium cell proliferation and migration. It inhibits methionine aminopeptidase 2 (MetAP-2) that is responsible for the cleavage of the N-terminal methionine residue from nascent proteins [4]. It is a drug with poor solubility and consequently its instability limits its potential for clinical translation, although some promising results have shown suppression of the inflammatory cytokine production *via* the local production of NO [5]. Other results suggest that MetAP-2 inhibition by fumagillin perturbed angiogenesis in zebrafish embryos [6].

It is well established that cardiovascular disease (CVD) is the main responsible of global deaths in the world. Atherosclerosis is a chronic systemic inflammatory disease characterized by the accumulation of several types of cells (macrophages, T cells, mast cells) and deposition of cholesterol-rich apolipoprotein B-containing lipoproteins within the subendothelial space in the arterial walls [7]. The most important consequences of atherosclerotic plaque rupture are myocardial infarction and stroke. Different imaging techniques have provided enough data that support the detection of atherosclerotic plaques at early stages in specific situations [8]. For example, the ability to specifically image macrophages with immunoliposomes may enable improved detection and characterization of atherosclerosis since the amount of enhancement observed by MRI is related to the macrophage content in the plaque [3,9]. Therefore, it seems that early detection of these vascular lesions is achievable in order to avoid complications derived from the atherogenic process. A key biological feature of the atherosclerotic process is the expansion of microvascular networks of vasa vasorum confined to the adventitia and outer media into the thickened intimal layer of the atheroma. Angiogenesis occurs in association with remodeling and protease activation in the surrounding tissues [10]. Consequently, several inhibiting compounds such as fumagillin, endostatin, PLP [11], thalidomide, U0126, and TGFβ [12] have shown to slow down the development of plaque formation and inflammation, as tested in animal models.

The aim of this study was to test the action of the anti-angiogenic agent fumagillin in early atherosclerotic lesions and to observe if the addition of the therapeutic drug fumagillin attenuates the growth of the atheromata. To carry out the study, three different types of contrast agents were prepared: plain liposomes (**L**), liposomes with fumagillin (**LF**), and immunoliposomes with fumagillin (**ILF**) including antibody CD36 for the targeting of macrophages.

## Material and methods

Phospholipids (1-palmitoyl-2-oleoyl-sn-glycero-3-phosphocholine (POPC), 1, 2-dipalmitoyl-sn-glycero-3-phosphoethanolamine-N-7-nitro-2-1, 3-benzoxadiazol-4-yl (DPPE-NBD), 1, 2-dipalmitoyl-sn-glycero-3-phosphoethanolamine-N-biotinyl (DPPE-Biotin), were purchased from Avanti Polar Lipids, Inc. (Alabaster, AL, USA). Solvents, CD36 antibody and fumagillin were acquired from Aldrich Chemical Co (St. Louis, MO, USA). The Spectra/Por membrane (Cellulose MWCO: 20,000 Da) was used for dialysis (Spectrum Medical Industries, Inc., Laguna Hills, CA, USA)

### Synthesis of liposomes

Liposomes were made from the phospholipid 1-palmitoyl-2-oleoyl-sn-glycero-3-phosphocholine (POPC), 1, 2-dipalmitoyl-sn-glycero-3-phosphoethanolamine-N-7-nitro-2-1, 3-benzoxadiazol-4-yl (DPPE-NBD), 1, 2-Dipalmitoyl-sn-glycero-3-phosphoethanolamine-N-biotinyl (DPPE-Biotin), a surfactant (Tween 80), and an aliphatic Gd complex (Gd-AAZTA-C17) with a molar ratio of 78.5/2/0.5/12/7. The lipid mixture was dissolved in a 1:1 chloroform/methanol solution (5 mL) and evaporated under nitrogen flux yielding a thin film that was then rehydrated. Thereafter, the lipid film was heated and sonicated twice for 15 min at 70W at 90% duty cycle. Since the liposome platform is very labile, a homing phospholipid that includes a biotin moiety was added for conjugation with biotinylated antibodies via avidin bridge. In order to increase the signals from MRI and fluorescence an antibody targeting the macrophage scavenger receptor-B (CD36) was attached to the liposomes. Gd-AAZTA-C17 was synthesized according to Gianolio *et al*.[13]

### Characterization of liposomes

Dynamic light scattering was performed on a Malvern instrument (Zetasizer, Nano-S Malvern Instruments, Westborough, MA) to determine the hydrodynamic diameter of a suspension of liposomes and reported as the mean Z-averaged diameter, and polydispersity index (PDI) from a cumulants analysis of three measurements. The number of gadolinium ions per liposome was determined by inductively coupled plasma optical emission spectroscopy (Maxxam Analytics, Burnaby, British Columbia, Canada) and this data was used to determine the number of gadolinium molecules per liposome.

### HPLC fumagillin determination

**Fig 2:**
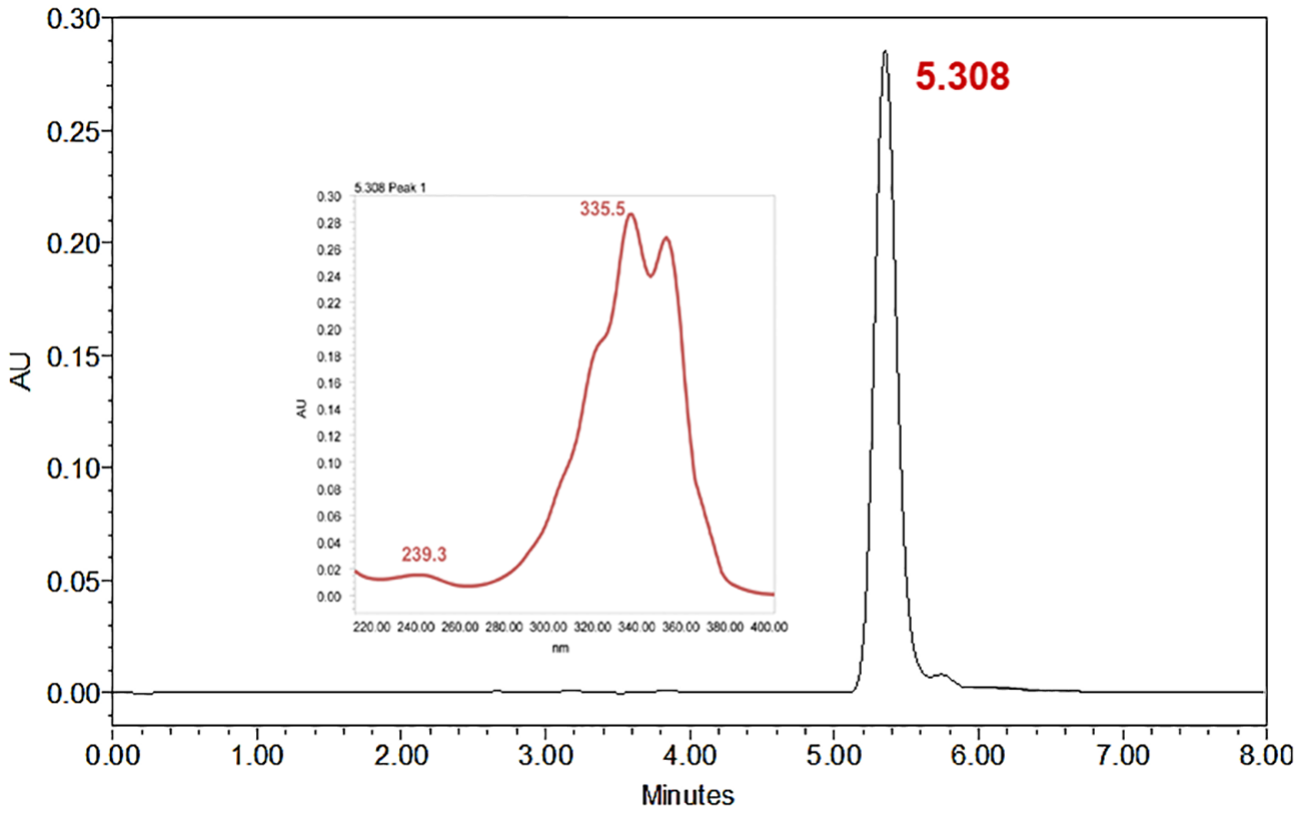
Representative chromatogram of the fumagillin sample obtained after liposome extraction. Fumagillin spectra presented two maximum peaks of absorbance at 335.5 and 351.1 nm. We used a wavelength of 351nm for the detection of fumagillin to avoid interferences from other liposome components. The retention time obtained was 5.35 ± 0.05 minutes (mean ± SD; n = 10).

Fumagillin loading was determined by HPLC analysis. HPLC of the liposome sample was run by triplicate using a NH_4_H_2_PO_4_:CH_3_CN (50:50 v/v, pH 4.8) mixture in a Kromasil^®^ C18 column (4.0 x 250 mm) at room temperature with a UV/VIS diode-array detector measuring absorbance set up at 351 nm (Chromatographic conditions are specified in Table 1 and the resulting chromatogram is shown in Fig 2). The synthesized liposomes used as therapeutic agents (**LF** and **ILF**) contained 24.8 μg of fumagillin/mL, which represents an average of 120 molecules of fumagillin per liposome [14].

**Table 1:** Chromatographic conditions for the analysis of fumagillin content by HPLC-UV.

Component	Chromatographic Conditions
Column	Kromasil^®^ (250 x 4.0 mm, 5 μm)
Mobile phase [A:B (v/v)]^a^	50:50
pH	4.8
Flow rate	1.0 mL·min-1
Injection volume	50 μL
UV detection	351 nm

^a^Mobile phase [A (ammonium di-hydrogen phosphate): B (acetonitrile)]

### Experimental animals and quantification of atherosclerosis burden

ApoE-KO engineering modified 8 week-old mice (Charles River Laboratories) were fed with an atherogenic high fat diet for 9 weeks (4 weeks prior to randomization to treatment groups and 5 weeks during treatment). Animal care and procedures were in accordance with institutional guidelines and regulations of Centro Nacional de Investigaciones Cardiovasculares (CNIC) and were approved by the Centro Nacional de Investigaciones Cardiovasculares Animal Care and Ethics Committee and regional authorities. Fat-fed mice were euthanized and aortas were removed after *in situ* perfusion with PBS followed by 4% paraformaldehyde/PBS. The entire aortas from the root to abdominal aorta, were dissected free, fixed overnight and stained with Oil Red O (0.2% Oil Red O in 80% MeOH, Sigma) [15]. An operator who was blinded to mouse genotype quantified the extent of atherosclerosis by computer-assisted morphometric analysis (SigmaScan Pro5, Aspire Software International, Ashburn, Virginia) of whole-mounted aorta.

### Magnetic resonance imaging experiments

*In vivo* imaging was performed with a Philips 1.5T system. Animals were anesthetized and scanned 24 hours after the injection of the different formulations (**L**, **LF**, and **ILF**). Four continuous axial images were obtained with T1 GEMS sequence. The imaging parameters were as follows: TR = 4 ms, TE = 2.34 ms, TI = 200 ms, and a flip angle of 35°. The images had a slice thickness of 1 mm, FOV 22 x 22 mm, matrix size 256 x 256, and a resolution of 0.0895 mm/pixel.

### Statistical analysis

Data were analyzed using SPSS software version 22 [16]. The inhibitory effects on plaque growth of the plain (without fumagillin) and theranostic liposomes (with fumagillin) were compared with the Mann-Whitney test. P values < 0.05 were considered statistically significant. Results are given as mean ±SD.

## Results

The above commented unique features of liposomes, such as their small size, biodegradability, biocompatibility, low toxicity, high carrying capacity, and ease of preparation and surface modification, make them excellent drug delivery vehicles [17]. Formulation included DPPE-NBD, a phospholipid that incorporates a fluorescent probe in its polar head, and the lipophilic complex Gd-AAZTA-C17 for fluorescent and MR imaging visualization, respectively. The Gd-AAZTA-C17 is a contrast agent based on a modified diazepine with a hydration number of q = 2 water molecules and renders high relaxivity when compared with typical based macrocycle or open chain polyamine agents [13,18]. An antibody able to target the macrophage scavenger receptor-B (CD36) was coupled to the surface of liposomes for specific delivery of immunoliposomes to the lesion. The diameter of the liposomes was determined by dynamic light scattering resulting in 98.7 nm with a PDI of 0.149.

**Fig 3:**
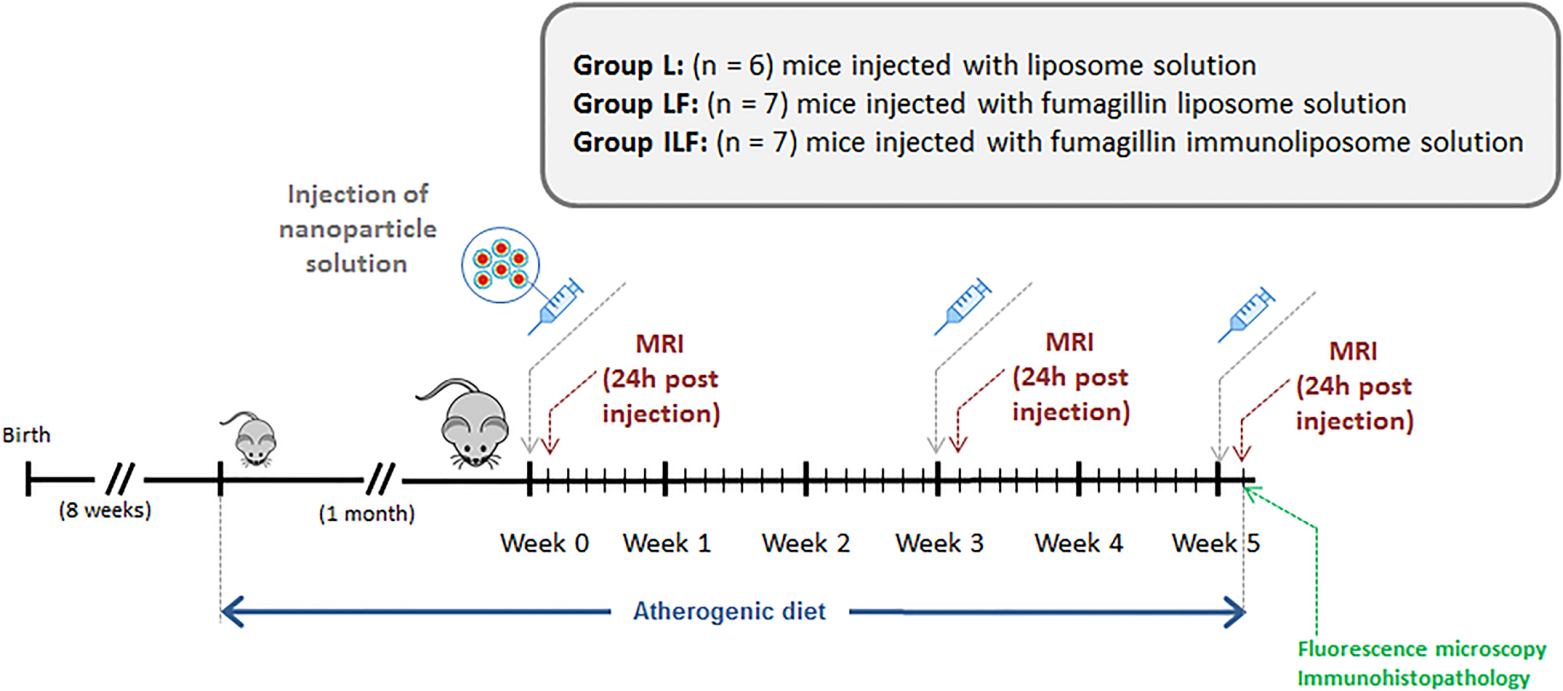
Experimental plan designed to test the action of liposomes loaded with the anti-angiogenic agent fumagillin in early atherosclerotic lesions.

In order to test the efficacy of the therapeutic nanoparticles, an experimental plan was aimed (Fig 3) in which the animals were fed with an atherogenic diet one month previous to the MRI studies and during the whole experimental period (weeks 0–5). Liposome-based contrast agents were intravenously injected on the first day of weeks 0, 3 and 5 (24.8 μg of fumagillin/mL). Since the maximum signal enhancement obtained with this type of agents is observed after 24 hours, the animals were scanned for MR imaging 24h post-injection of the nanoparticles. Gadolinium concentration in liposomes was assessed by inductively coupled plasma optical emission spectrometry (ICP-OES). The concentration of gadolinium in each type of liposome resulted in an average of 5400 molecules of Gd-AAZTA-C17 per liposome. This value represents a dose of 7 μmol of Gd/kg for an injection volume of 125 μL.

Three experimental groups were defined to assess the efficiency of the therapeutic liposomes in the detection and treatment of early atherosclerotic lesions. Animals in the first group (**L**) received plain liposomes solution as a control (n = 6). The mice of the second group (**LF**) received an injection of liposomes carrying the anti-angiogenic drug fumagillin (n = 7). The animals of the third group were injected with immunoliposomes loaded with fumagillin (**ILF**) (n = 7). Each mouse received 125 μL of nanoparticle suspension. Animals tolerated the injections and showed regular weight gain, activity levels, and no ulceration at the injection sites.

At the end of the study, the animals were euthanized and the aortas harvested and imaged with Xenogen IVIS system for fluorescence imaging. *En face* preparations were digitally photographed and then stained with Oil Red O for histochemical studies. Morphometric analysis of scanned images was performed using Sigma Scan Pro5 software and the percentage of total area of the arch and thoracic aorta covered by plaque was calculated. At the time of euthanasia, there were no difference in body weight between animals in different experimental groups.

**Fig 4:**
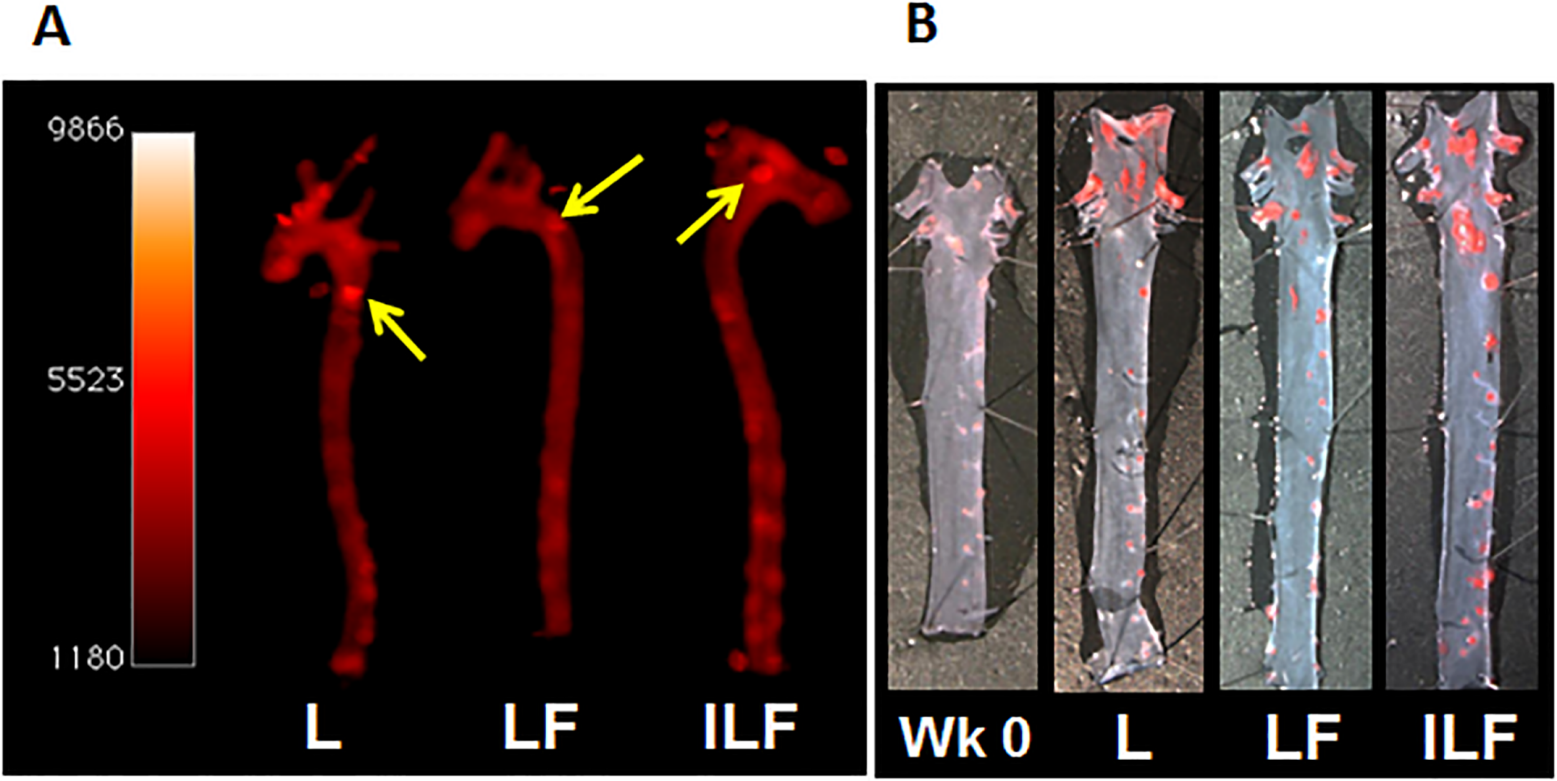
(A) Fluorescence imaging of harvested aortas. The arrows point the patchy areas where plaques are located. (B) Harvested aortas stained with Oil Red O.

The fluorescent and microscopy images of the stained aortas revealed the presence of scattered patchy small lesions mainly located in the aortic arch with scattered small hotspots in the abdominal aorta (Fig 4). These hotspots were present in all experimental groups (**L**, **LF** and **ILF**) and correlated with plaque development, showing the efficiency of our liposomes to reach atherosclerotic lesions. The lesion area of aortic atherosclerosis was expressed as the percentage of Oil Red O-stained area relative to the area of the aortic arch.

We evaluated the effect of the theranostic nanoparticles carrying the drug fumagillin (**LF** and **ILF**) on inhibiting plaque growth. Since this compound is very insoluble in aqueous media, our purpose was to incorporate it within liposomes to deliver fumagillin to the plaques. For comparison, we included a vehicle control group that received plain liposomes (**L**) to observe the atherosclerotic lesion progression in the absence of any treatment. In addition, we also adjusted for the baseline level of plaque in mice after one month feeding with atherogenic diet and prior to any treatment (**Wk 0**). The areas of atherosclerotic lesions were markedly increased in the **L** group when compared with the **LF** and **ILF** treated mice. Quantitative analysis was performed by a trained operator blinded to mouse treatment. Mean atherosclerotic lesion areas were determined using computer-assisted image analysis (values reported in Table 2).

**Table 2:** Percentage of lesion area relative to the area of the aortic arch.

	Mean percent aortic plaque area	Plaque inhibition	P
**Wk 0** (n = 6)	1.6 ± 0.4	-	-
**L** (n = 6)	18.01 ± 0.97	-	-
**LF** (n = 7)	13.1 ± 1.5	23.7%	0.037
**ILF** (n = 7)	15.5 ± 3.2	4%	0.522

**Fig 5:**
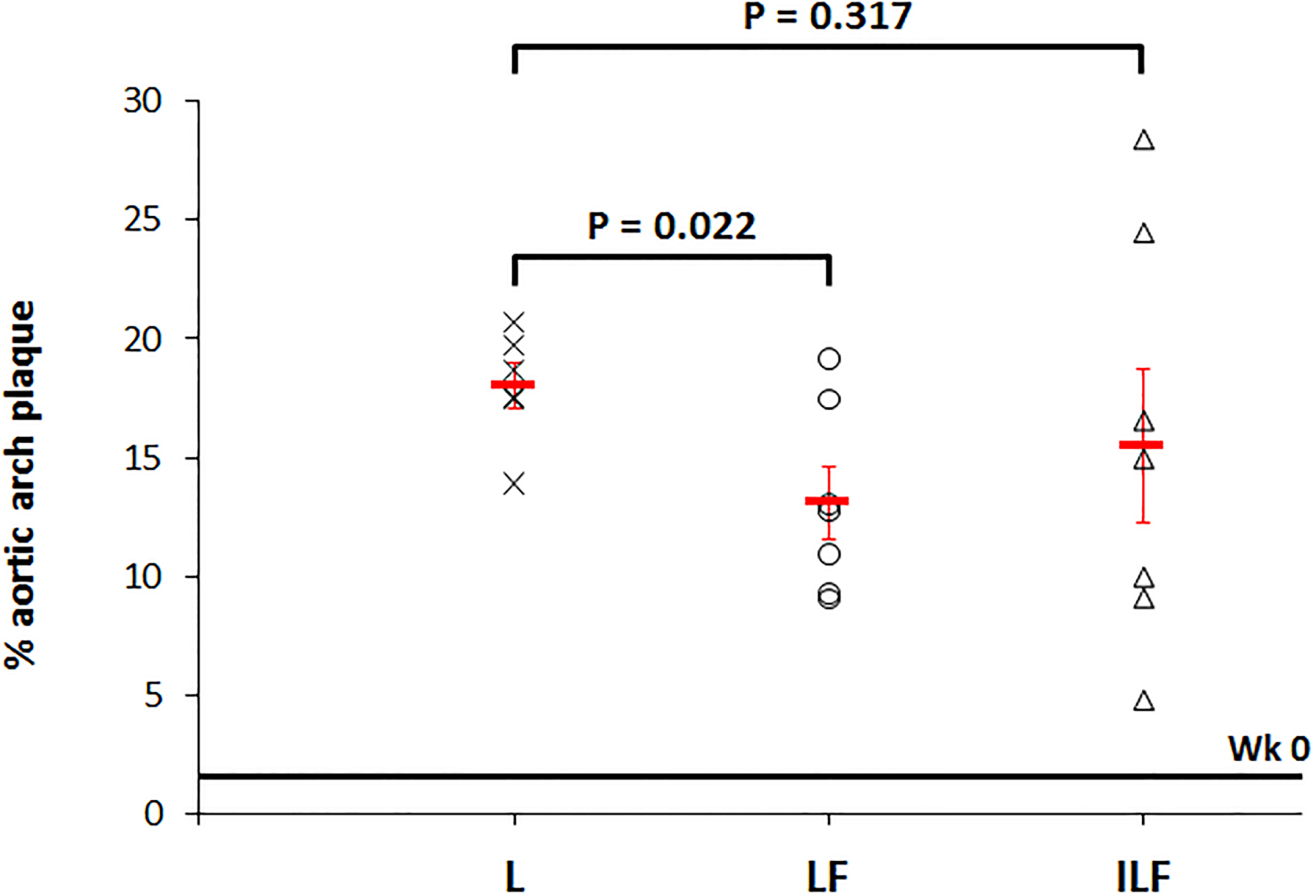
Percent of aortic arch plaque obtained after treatment with angiogenesis inhibitors (LF and ILF) in mice. Plain liposomes control **L** (x), liposomes loaded with fumagillin **LF** (○), and immunoliposomes with fumagillin **ILF** (Δ) animals were treated for 5 weeks as previously described. Red line centered at each group represents median plaque area of aortic arch lesions measured in a cohort (n = 6).

The median percent aortic plaque areas for mice at baseline (**Wk 0**) was 1.6 ± 0.4 (n = 6). This value is represented by a straight line in Fig 5 and provides a general comparison point from which to determine the development and progression of atherosclerosis. The injection of plain liposomes (**L**) allowed the normal progression of the disease during 5 weeks with a mean percent aortic plaque area of 18.01 ± 0.97 (n = 6). After treatment with fumagillin, lesions developed in the aortic arch showed significant decrease in the case of **LF** when compared with **L** (13.1 ± 1.5 (n = 7); P = 0.037), while the treatment with **ILF** revealed no significant differences (15.5 ± 3.2 (n = 7); P = 0.522).

Since the mean area of lesions without treatment was 18.01 ± 0.97, short-term treatment with **LF** appeared to inhibit plaque growth by 23.7% while the same treatment with fumagillin loaded targeted liposomes (**ILF**) resulted in only a 4% inhibition when compared with the liposome control group. Percent inhibition of plaque growth by fumagillin nanoparticles was calculated according to Eq (1) [10]

$$\%\;\text{inhibition}=100*\left[1-\left(\frac{\text{median}\;\text{percent}\;\text{plaque}\;\text{area}\;\text{treated}-0.25}{\text{median}\;\text{percent}\;\text{plaque}\;\text{area}\;\text{control}-0.25}\right)\right]$$ (1)

**Fig 6:**
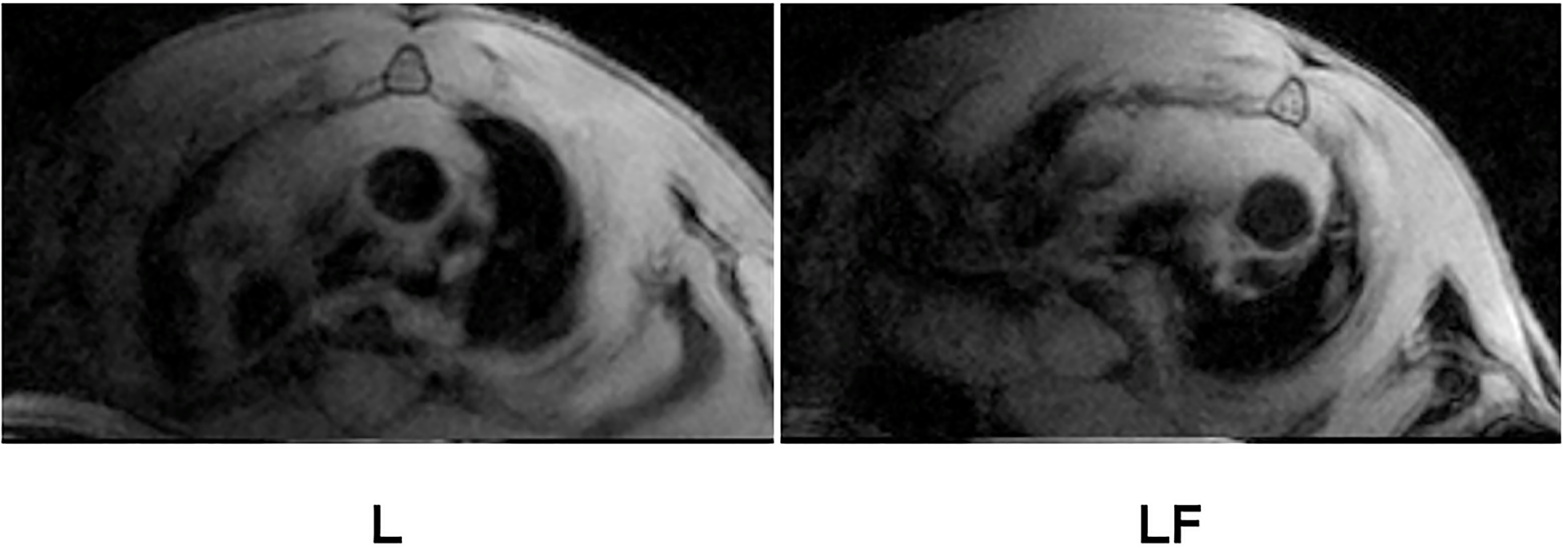
Cross-sectional images of the aortic arch on *in vivo* T1-weighted MRI of mice treated with L and LF liposomes and obtained 24 hours after final injection.

*In vivo* MRI of the aortic arch in all animals performed after last injection of liposomes showed unnoticeable luminal narrowing and wall thickening (Fig 6). This result was confirmed by quantitative analysis through planimetry and fluorescent imaging, evidencing patchy areas in the aortic arch and even more scatter small lesions in the thoracic and abdominal aorta. Immunoliposomes did not improve signal intensity of the lesion since the antibody chosen for targeting the plaque is directed against the macrophage scavenger receptor B and, at this early stage, the content of macrophages in atheromata is low.

## Discussion

The results obtained with our liposome nanoparticles loaded with fumagillin (**LF**) compared with control liposomes (**L**), suggest that **LF** liposomes are capable to inhibit plaque development to a similar percent (23.7%) obtained in the experiment performed by Moulton using the fumagillin derivative TNP-470. Nevertheless, our approach aimed to investigate the effect of fumagillin liposomes on early atherosclerotic lesions (ApoE-KO mice at 12–17 weeks of age). Our study was conducted for a shorter period (only 5 weeks compared to 16 weeks of treatment carried out by Moulton) and lower doses of the anti-angiogenic drug were injected only three times during the whole experiment (compared to higher doses injected each other day during 16 weeks in Moulton study.

Although fatty streaks normally appear at 8–20 weeks of age in ApoE-KO mice fed with an atherogenic diet, and plaques with smooth muscle cells are initially observed at 15 weeks, our **LF** nanoparticles accumulated in early stage atherosclerotic plaques confirmed by fluorescence imaging and histochemical analysis.[19,20]. Images revealed atherosclerotic plaques in the arch region, as well as small lesion development in the thoracic aorta. Therefore, **LF** enables early treatment of atherosclerotic lesions containing little intimal neovascularization.

The use of antibodies that bind to mononuclear cells such as monocytes, macrophages, and foam cells may be a means of potentially increasing targeting efficiency of atherosclerosis since these cells have been shown to play a key role in the progression of disease [21]. CD36 is a glycoprotein that is present in the membrane of macrophages, thus antiCD36 targets the macrophage scavenger receptor-B facilitating the *in vivo* imaging of atherosclerotic plaques according to the amount of macrophages accumulated in the lesion. This was demonstrated by Lipinski *et al* showing signal enhancement in advanced plaque imaging studies [19]. Taking this into consideration, we synthesized immunoliposomes including antiCD36 loaded with fumagillin (**ILF**) for better targeting and therapeutic efficiency. However, **ILF** produced no significant inhibition compared with control liposomes (**L**), which can be attributed to the very early stage of the plaques. In our *in vivo* experiment, the small percentage of atherosclerotic lesions present in the aortic arch just reflected the low macrophages content in the lesion. Treatment with immunoliposomes resulted in poor targeting and consequently in little decrease of plaque development. This was evidenced with a smaller inhibition percentage than in the case of non-targeted liposomes (**LF**). Additionally, it must be taking into consideration that intravenous administration of nanoparticles able to target CD36 protein can be retained in the liver by binding to scavenger receptors expressed on the surface of Kuppfer cells [22] or to any other cells that express CD36 such as endothelial cells, smooth muscle cells, adipocytes, and platelets.

Regarding to the choice of the therapeutic agent, the important role of angiogenesis in atherosclerosis has generated wide interest during the last years since many studies suggest that angiogenesis constitutes a potential target for the treatment of atherosclerosis [23,24]. Novel and specific therapies based on the administration of anti-angiogenic agents have shown to decrease both neovascular proliferation and plaque development in animal models of atherosclerosis [25,26]. Effective inhibitors of angiogenesis include fumagillin and synthetic derivatives. Fumagillin has proven to exhibit anti-angiogenic effects in cancer therapies [27,28], and more recently research has shown that the administration of TNP-470, a water soluble form of fumagillin, resulted in significant reduction in plaque growth [10]. However, there was no significant effect at the very early stages of atherosclerotic plaque formation and furthermore, systemic administration of high doses of TNP-470 during prolonged period of time is associated with neurotoxic effects [29].

For the purpose of diminishing side effects, we have developed targeted (**ILF**) and non-targeted (**LF**) fumagillin loaded liposomes as theranostic strategy against plaque progression. Since fumagillin is highly hydrophobic, the compound will probably be entrapped within the phospholipid bilayer of the nanoparticle. Fumagillin is probably delivered into the cells by a mechanism referred to as “contact-facilitated drug delivery” (CFDD), a process in which the liposome containing the therapeutic drug facilitates the interaction and hemifusion with the target cell surface phospholipids promoting the passive transfer of the drug [12].

## Conclusion

We have developed liposome-based nanoparticles able to reach atherosclerotic lesions. Anti-angiogenesis treatment with fumagillin-loaded liposomes appears to inhibit atherosclerotic plaque growth, suggesting that even early treatment can significantly attenuate the development of atherosclerosis. The use of antiCD36 immunoliposomes did not significantly inhibit the development of atherosclerosis compared with a naked liposome control group.

## Supporting information

S1 Table Percent of aortic arch plaque data. (PDF)

S1 Fig Graphical abstract figure. (TIF)
