# Supplementary material for: Anti-angiogenic drug loaded liposomes: Nanotherapy for early atherosclerotic lesions in mice
Source: PLoS One. 2018 Jan 16;13(1):e0190540. doi: 10.1371/journal.pone.0190540 (PMC5770017; doi:10.1371/journal.pone.0190540)
Supplement: S1 Table — (PDF) [file pone.0190540.s001.pdf]

S1 Table. Percent of aortic arch plaque data.

Liposomes	Percent aortic arch plaque
**L**	13,91
**L**	17,45
**L**	17,55
**L**	18,71
**L**	19,72
**L**	20,71
**LF**	9,08
**LF**	9,29
**LF**	10,96
**LF**	12,76
**LF**	13,06
**LF**	17,44
**LF**	19,17
**ILF**	4,82
**ILF**	9,11
**ILF**	10
**ILF**	14,97
**ILF**	16,58
**ILF**	24,47
**ILF**	28,39
