# Supplementary figures and images for: Anti-angiogenic drug loaded liposomes: Nanotherapy for early atherosclerotic lesions in mice
Source: PLoS One. 2018 Jan 16;13(1):e0190540. doi: 10.1371/journal.pone.0190540 (PMC5770017; doi:10.1371/journal.pone.0190540)

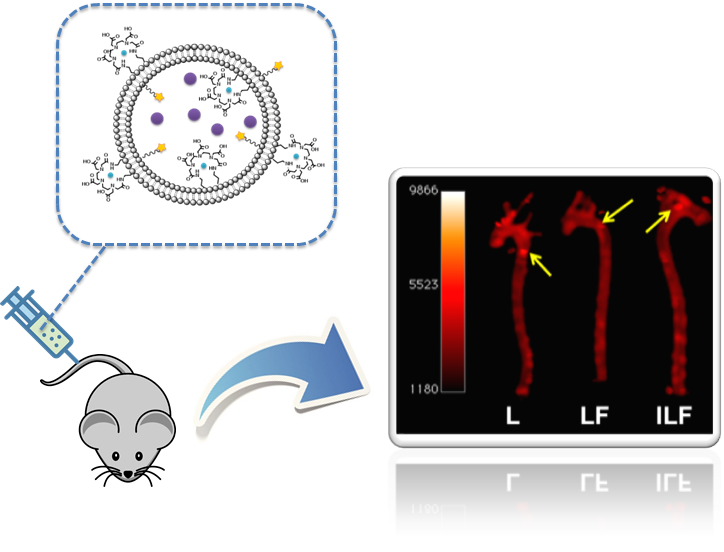

Supplement: S1 Fig — (TIF) [file pone.0190540.s002.tif]
